# *Citrus tristeza virus* Promotes the Acquisition and Transmission of ‘*Candidatus* Liberibacter Asiaticus’ by *Diaphorina citri*

**DOI:** 10.3390/v15040918

**Published:** 2023-04-04

**Authors:** Longtong Chen, Yangyang Liu, Fengnian Wu, Jingtian Zhang, Xiaoqing Cui, Shitong Wu, Xiaoling Deng, Meirong Xu

**Affiliations:** 1 Guangdong Province Key Laboratory of Microbial Signals and Disease Control, Citrus Huanglongbing Research Laboratory, South China Agricultural University, Guangzhou 510642, China; 2 School of Life Sciences and Food Engineering, Hanshan Normal University, Chaozhou 521041, China

**Keywords:** Huanglongbing, citrus, citrus tristeza disease, Asian citrus psyllid, co-infection

## Abstract

*Diaphorina citri* Kuwayama (*D. citri*) is an insect vector of phloem-limited ‘*Candidatus* Liberibacter asiatus’ (CLas), the presumed pathogen of citrus Huanglongbing (HLB). Recently, our lab has preliminarily found it acquired and transmitted *Citrus tristeza virus* (CTV), which was previously suggested to be vectored by species of aphids. However, the influences of one of the pathogens on the acquisition and transmission efficiency of the other pathogen remain unknown. In this study, CLas and CTV acquisition and transmission by *D*. *citri* at different development stages under field and laboratory conditions were determined. CTV could be detected from the nymphs, adults, and honeydew of *D*. *citri* but not from the eggs and exuviates of them. CLas in plants might inhibit CTV acquisition by *D*. *citri* as lower CTV–positive rates and CTV titers were detected in *D*. *citri* collected from HLB-affected trees compared to those from CLas–free trees. *D*. *citri* were more likely to obtain CTV than CLas from host plants co-infected with the two pathogens. Intriguingly, CTV in *D*. *citri* facilitated the acquisition and transmission of CLas, but CLas carried by *D*. *citri* had no significant effect on the transmission of CTV by the same vector. Molecular detection and microscopy methods confirmed the enrichment of CTV in the midgut after a 72-h acquisition access period. Collectively, these results raise essential scientific questions for further research on the molecular mechanism of pathogen transmission by *D*. *citri* and provide new ideas for the comprehensive prevention and control of HLB and CTV.

## 1. Introduction

Citrus Huanglongbing (HLB) is a bacterial disease caused by the fastidious “*Candidatus* Liberibacter spp.” [1]. It is one of the most devastating diseases in the worldwide citrus industry [2]. The citrus plants affected by HLB gradually lost their economic value due to fruit malformation, leaf yellowing, root rot, rapid tree decline, and finally, reduction of the fruit quantity and quality. Of the reported “*Ca*. L. spp.”, “*Ca*. L. asiaticus” (CLas) is the most prevalent and predominant species [3]. It is a significant scientific challenge toward effective HLB management because CLas is a noncultured phloem-inhabiting bacterium and is readily graft-transmissible and easily transmitted by its insect vector.

*Diaphorina citri* Kuwayama (Hemiptera) (*D. citri*) causes damage to most plants within the family Rutaceae [4], especially citrus. Both adult and nymph *D. citri* insects feed on the tender shoots and mature leaves of the host plants, which causes gradual withering of the tender shoots and the curling and falling of leaves, thus inhibiting the growth of the host plants. In addition, *D. citri* excretes sticky white honeydew after feeding, which leads to sooty mold in host plants and affects plant photosynthesis [5]. In the HLB epidemic areas, the indirect harm caused by *D. citri* as the vector transmitting CLas is significantly heavier than the direct harm caused by feeding [2]. In the field, 9–16 overlapping generations of *D*. *citri* can occur in a year. Adult females may lay more than 800 eggs during their lives of 14–49 days, depending mainly on the seasonal climatic conditions [6]. A single *D*. *citri* adult can acquire CLas from HLB–affected trees within a 24 h acquisition access period (AAP) and transmit it within a 1.5 h inoculation access period (IAP) [7,8,9]. The nymph *D. citri* insects have a significantly higher CLas acquisition rate than adult insects [8]. A range of 60–100% *D. citri* nymphs were CLas–positive after a 5-week AAP, and the rate for adult insects was up to 40% [10]. The 4th to 5th instars and adults of *D. citri* can carry CLas and transmit HLB in their whole life, but transovarial occurs at a rate of 2–6% [11].

*Citrus tristeza virus* (CTV) causes citrus tristeza disease, which is the most widely distributed viral disease in citrus production [12]. Different strains of CTV caused three main types of symptoms in citrus: quick decline (QD), stem pitting (SP), and seedling yellows (SY) [13]. In citrus production, resistant rootstocks and cross-protection induced by different CTV isolates are widely used to alleviate the harm of citrus tristeza disease. However, SP strains and QD virulent strains of CTV have been causing a severe reduction in citrus production when sour orange (*Citrus aurantium* L.) was used as rootstock or some popular cultivars like sweet orange (*Citrus sinensis* (L.) Osbeck) and grapefruit (*Citrus paradisi* MacFadyen) were widely planted [14,15]. The effective vector insects of CTV are suggested to be several species of aphids, mainly including *Toxoptera citricida*, *Aphis gossypii*, *A. citricola*, *T*. *aurantia*, and so on [14]. The mode of CTV transmission by aphids is non-cyclic and semi-persistent [16]. CTV could be obtained by *T. citridus* from citrus within 1–24 h AAP, with a persistence time of 24–48 h and a transmission time of 30 min–100 h [17]. The ability of CTV transmission and acquisition increased with the feeding time. Viruliferous aphids such as *T*. *citridus* and *A. gossypii* lost the ability to transmit CTV after 24 h of transmission to healthy citrus and became non-toxic aphids [18]. When comparing the transmission efficiency of a single CTV-carrying aphid onto sweet orange plants, the transmission rate of a single *T*. *citridus* was the highest (16%), followed by that of *A. gossypii* at 14%. Other aphids have even lower transmission rates [19,20].

It is common for a specific insect vector to be infected by different pathogens in nature; for example, small brown planthopper can transmit reoviruses (*rice black*–*streaked dwarf virus* and *Maize rough dwarf virus*) [21,22], tenuiviruses (*rice stripe virus*) [23], rhabdoviruses (*barley yellow striate mosaic virus* and *northern cereal mosaic virus*) [24,25], and cripaviruses (*Himetobi P virus*) [26]. Some other vectors would transmit different kinds of plant pathogens, e.g., the leafhopper *Inazuma dorsalis* transmits *Rice dwarf virus* and phytoplasma of Rice orange yellow leaf disease. Only a few insect vectors can transmit both plant-pathogenic bacteria and viruses. For example, *Circulifer tenellus* can transmit *Beet curly top virus* and two prokaryotes (‘*Candidatus* Phytoplasma trifolii’ and *Spiroplasma citri*) [27]. In insect vectors, different involved pathogens may be cooperative, neutral, or antagonistic [28] in their interaction. Wang et al. [29] found that brown planthoppers that carried *Rice ragged stunt virus* preferred *Southern rice black*–*streaked dwarf virus*-infected rice, resulting in co-infection with more than one virus in vectors and host plants. Furthermore, one pathogen in a vector insect can affect the replication of another. For example, the concentration of *Tomato yellow leaf curl virus* (TYLCV) increased after *Rickettsia rickettsii* infected the midgut of the whitefly, which increased the transmission efficiency of TYLCV [30]. *Mal de Río Cuarto virus* (MRCV) replication was inhibited in wheat *rhabdovirus*-carrying *Delphacodes kuscheli*, but the transmission capacity of MRCV by *Delphacodes kuscheli* was not affected [31].

Britt et al. [32] used high-throughput sequencing technology to detect the co-existence of multiple viruses in the population of *D. citri* in Florida, USA. They found that CTV was widely present in the field of a *D. citri* population with high abundance. In recent years, our lab firstly found a complete CTV genome in the *D. citri* cDNA sequence and further suggested that *D. citri* can acquire, carry, and transmit CTV [33].

To understand more of the CTV transmission mode by *D. citri*, the influence of CTV or CLas infection of the *D. citri* on the acquisition, retention, and transmission of the other pathogen, as well as how interactions between CLas and CTV co-infected citrus plants may influence the transmission of another pathogen, we conduct this study in both field and laboratory conditions. Results illustrate that competition by CTV may change accumulation patterns of CLas within the vector and influence the *D. citri* transmission efficiency of CLas.

## 2. Materials and Methods

### 2.1. Plant Materials

Citrus material in the field: samples were collected from three HLB-affected orchards with different citrus cultivars in Zhanjiang and Deqing cities of Guangdong province, China. Ten to sixteen citrus trees with CTV infection (confirmed by RT-qPCR) and with *D. citri* feeding on were selected from each orchard (Table A1). Four leaf shoots from each citrus tree in the directions of east, west, south, and north, respectively, were selected and kept on ice. Leaves selected from these shoots (one leaf from each shoot) were quickly cleaned with sterilized ddH_2_O and stored immediately in liquid nitrogen. Midribs of the four leaves were cut and mixed for subsequent RNA extraction and pathogen (CLas and CTV) detection.

Plant materials in laboratory conditions: Plants for rearing *D. citri* population were 2-year-old healthy orange jasmine (*Murraya paniculata*) seedlings and 2-year-old healthy *Citrus reticulata* Blanco “Shatangju” seedlings. Scions carrying CLas (A4 strain) and/or CTV (CT31 strain) were side-grafted to the “Shatangju” rootstocks to generate plants with different pathogens. After graft–inoculation, the tops of canopies were pruned to stimulate the even distribution of pathogen(s). Six months after grafting, the infection status of the trees was confirmed by RT–qPCR. The information on plants used for CLas or CTV acquisition by *D. citri* is shown in Table 1. Twelve healthy “Shatangju” plants were used for CTV and/or CLas transmission by *D. citri*.

Plants were kept in the greenhouse ambient with seminatural light and temperature conditions until being transferred to the growth chambers when necessary. The plants were watered every other day, fertilized using compound fertilizer biweekly, and pruned monthly to generate sufficient new shoots. Conditions in the secure quarantine facilities (growth chambers) for plant and psyllid colony maintenance were set as follows: illumination for 14 h at a temperature of 27 ± 1 °C, dark for 10 h at 25 ± 1 °C and the relative humidity (RH) of 70–80%. All plants were initially screened by RT–qPCR using specific primers and ELISA (direct triple antibody sandwich protocol, TAS–ELISA) to be free of CTV, CLas, and other common pathogens, e.g., *Citrus exocortis viroid*, *Citrus chlorotic dwarf-associated virus*, *Citrus tatter leaf virus*, and *Citrus yellow vein clearing virus* [34]. Antibody (Catalog No: CAB 78900; Lot No: 00805) specific to the CTV capsid protein was purchased from Agdia Company (Elkhart, IN, USA).

### 2.2. Diaphorina citri Samples

*Diaphorina citri* collected from citrus orchards were all from the above-mentioned trees from three orchards (Table A1). A sufficient number of *D. citri* at different ages were collected from four branches of each citrus tree in four directions (Table A2). All *D. citri* samples from a tree were kept in a collection tube separately and temporarily maintained at low temperatures for subsequent RNA extraction and pathogen (CLas and CTV) detection individually in the lab.

Breeding and feeding conditions of *D. citri* colony: Adult *D. citri* were collected from the orange jasmine plants on the campus of SCAU. After one-quarter of them were randomly selected and detected to be free of CLas and CTV, the other *D. citri* were raised on healthy orange jasmine plants in a temperature-controlled chamber. Twenty *D*. *citri* were tested (with 2 adults and 2 nymphs mixed as one sample) by RT–qPCR biweekly to ensure the colony was free of CTV or CLas. When *D. citri* had multiplied to more than 10 generations, the population of *D. citri* was further used in the following experiments.

For CTV and/or CTV acquisition and transmission experiments, the third–instar *D. citri* nymphs or 7-day-post-emergence adult insects were collected and enclosed in meshed plastic gauze bags of 250 mm × 180 mm on branches without touching the leaves. Populations of *D. citri* carrying CTV and/or CLas were generated by rearing the third–instar nymph *D. citri* insects on ‘Shatangju’ plants carrying CLas and/or CTV (CLas^+^CTV^+^, CLas^+^CTV^−^, and CTV^+^CLas^−^) and healthy (CTV^−^CLas^−^) “Shatangju” trees for days, and recorded as CLas^+^CTV^+^, CLas^+^CTV^−^, CTV^+^CLas^−^, and CTV^−^CLas^−^ *D. citri* populations, respectively. Thereafter, the emerged *D. citri* adults were used in the acquisition and transmission experiments.

### 2.3. RNA Extraction and cDNA Synthesis

Total plant RNA was extracted from the midrib of either citrus leaves or orange jasmine leaves using plant tissue E.Z.N.A.^®^ HP plant RNA extraction kit (Omega Bio–tek., Norcross, GA, USA). Genomic DNA contamination was eliminated by digestion with RNase-free DNase I (TaKaRa Bio–tek., Shuzo, Kyoto, Japan). Total insect RNA was extracted from tissues of a single *D. citri* using TRlzol^®^ Reagent (Life Technologies, Guangzhou, China). The concentration and purity of total RNA were determined by absorbance using NanoDrop™ One (Thermo Scientific, Shanghai, China). RNA samples with OD260/OD230 of 2.0–2.4 and OD260/OD280 of 1.8−2.2 were selected and stored at −80 °C for further use. The total RNA samples were individually used for reverse transcription by Verso cDNA Synthesis (TransScript) kit (TransGen Biotech, Beijing, China).

### 2.4. Quantification of CTV and CLas by RT–qPCR

The primers used to detect CTV and CLas were named cquctv1/2, designed based on the *p20* gene of CTV [35], and named CLas4G/HLBr based on the three copies of the *16srRNA* genes [36], respectively. RT–qPCR was performed using Bestar^®^ DBI SYBR Green PCR Reagent Kits (DBI Bioscience, Shanghai, China) according to the manufacturer’s instructions. The 20 μL reaction system consists of 10 μL of SYBR Green Mix, 8 μL of dd H_2_O, 0.5 μL of forward and reverse primers (10 pM), respectively, and 1 μL of DNA (cDNA). RT–qPCR reaction conditions were set as pre-denaturation at 95 °C for 2 min, 40 cycles of denaturation at 95 °C for 15 s, and extension at 60 °C for 20 s. cDNA samples with Ct values less than 33 were considered CTV-positive. Concomitantly, to relatively quantify the pathogen titers, standard curves were drawn using pEASY–T1 (TransGen Biotech, Beijing, China) recombinant cloning plasmids containing the target fragments and the relative primers, with 8 gradients (10(7)-fold). CTV and CLas concentrations were assessed by the copy number of CTV or CLas in per ng cDNA using a formula referencing to the study of Ruiz–Ruiz et al. [15].

### 2.5. Sample Collection and Experimental Designs under Laboratory Conditions

All plants and psyllid used in AAP and IAP were maintained in plant growth chambers under the conditions mentioned above.

CLas and CTV acquisition by *D. citri* in trees co-infected by the two pathogens: This experiment was carried out after confirming the eggs of *D. citri* from the field’s CTV-positive (detected by RT–qPCR) trees were without detectable CTV. More than 100 healthy adult *D. citri* (with a male-to-female ratio of about 1:1) were collected into a mesh bag and transferred onto the young shoots of “Shatangju” plants carrying both CLas and CTV (CLas^+^CTV^+^) for AAPs. After laying a large number of eggs on the buds, adult *D. citri* insects were removed. The eggs hatched gradually until the number of different stages of nymph and adult insects was more than 30 on the plants. Forty individuals of each 3rd, 4th, and 5th instars, 10 1-min-old postemergence (emerged on CTV^−^ “Shatangju” plants) adults, and 24 6-hour-old postemergence adult insects (moved to the healthy ‘Shatangju’ plants for emergence) were collected respectively for pathogen detection.

CTV acquisition by *D. citri* with and without CLas: This experiment was done with six replications. According to the study of Inoue et al. [7] and Pelz–stelinski et al. [8], *D. citri* nymphs were used to acquire the CLas. More than 70 healthy third–instar nymphs were transferred onto the young shoots of CLas^+^CTV^−^ *Citrus tangerine* Hort. plants for a 15-day AAP to acquire CLas. On the 7th and 14th days of bagging rearing, five *D. citri* were collected from each plant for CLas detection individually. At 15-day AAP, adult *D. citri* populations (about 7-day postemergence) with more than 50% individuals carrying CLas (named CLas^+^CTV^−^) were obtained. Meanwhile, more than 70 healthy third–instar nymphs were placed on a healthy tree and bagged for 15 days’ rearing as control (named CLas^−^CTV^−^ population). Both the emerged CLas^+^CTV^−^ population and the CLas^−^CTV^−^ population were enclosed in meshed plastic gauze bags and transferred to 6 CTV^+^CLas^−^ *Citrus grandis* Osbeck plants for another 10-day AAP to acquire CTV. Ten adult CLas^+^CTV^−^ insects or 10 adult CLas^−^CTV^−^ insects were assigned to each plant in a bag. On the 1st, 3rd, 7th, and 10th day after CTV AAP, 10 *D. citri* adults from each population were randomly collected from the plants, and RNA was extracted individually for the pathogen (CLas and CTV) detection. The CTV acquisition efficiency and CTV titers were compared between the two *D. citri* populations with and without CLas.

CLas acquisition by *D. citri* with and without CTV: Likewise, the CLas^−^CTV^+^ psyllid population was generated in *Citrus grandis* Osbeck plants and subsequently used to compare the CLas acquisition efficiency by *D. citri* with and without CTV. Both the CLas^−^CTV^+^ and the CLas^−^CTV^−^ psyllid populations were transferred to CTV^−^CLas^+^ *Citrus tangerine* Hort. Ex Tanaka plants for CLas acquisition. *D. citri* samples were collected for pathogen detection.

CLas and CTV transmission by different *D. citri*: Four psyllid populations (more than 120 psyllids in each population), namely the CLas^−^CTV^+^, CLas^+^CTV^−^, CLas^+^CTV^+^, and the CLas^−^CTV^−^, were generated separately from the corresponding citrus plants and healthy orange jasmine seedlings, respectively. Three experimental replications were conducted. Specifically, more than 40 healthy third–instar nymphs were reared on each plant enclosed by meshed plastic gauze bags. On the 14th day of AAP, 10 *D. citri* were randomly collected from each plant for RNA extraction and pathogen (CLas and CTV) detection individually. The other 30 psyllids in each population were transferred to 3 healthy *Citrus reticulata* Blanco “Shatangju” plants that were in good growing conditions and with sufficient new shoots, with 10 insects on each plant, to transmit CTV and/or CLas or used as control. To avoid the influence of secreted honeydews on pathogen detection, all *D. citri* and eggs, together with the enclosing bags, were removed from the 12 “Shatangju” plants after 15-days-IAP. At this time, plants were recorded as 0 days post inoculation (0 dpi) and were transferred to the insect-proof screenhouse. Fully expanded new leaves of approximately the same age were sampled at 0 dpi, 15 dpi, 30 dpi, 45 dpi, 60 dpi, and 150 dpi for RNA extraction and pathogens detection. Three leaves from each of the three shoots, with one fed by the psyllids, from each plant were sampled. One leaf equals one replicate in the pathogen detection. The symptoms of “Shatangju” trees were photographed at the same time. The transmission effects were defined according to the RT–qPCR results and symptoms.

### 2.6. The Acquisition and Persistence Mode of CTV by D. citri

The complete midguts and salivary glands of 90 (3 replications) adult *D. citri* insects from the population with 66.67% CTV-positive rate were individually dissected by using Insect anatomy stereo microscope Gmbh 37,081 (Carl Zeiss Jena GmbH, Gottingen, Germany) and 0.05 mm dissection forceps. RT–qPCR was used to detect the CTV titers in these two parts. After confirming the enrichment of CTV in gut tissues, midgut samples from psyllid on CTV-positive trees were further used to investigate the CTV propagation status within three days’ AAP. Specifically, more than 300 adult *D*. *citri* were collected from orange jasmine trees and enclosed in mesh bags for 10 h of starvation treatment. Then CTV acquisition was evaluated by confining three groups of 100 adult *D*. *citri* on CT31–infected ‘Shatangju’ shoots (Ct = 21.68 ± 1.93). Ten *D*. *citri* insects on 0 h AAP, 6 h AAP, 24 h AAP, 48 h AAP, and 72 h AAP were collected separately and subsequently dissected individually to obtain the midgut (*n* = 3 × 10 psyllids per AAP). CTV titers in the midgut tissues were evaluated by RT–qPCR. For the CTV persistence assay, groups of the CTV^+^ *D*. *citri* adults (with more than 80% individuals infected) were transferred onto CTV^−^ orange jasmine trees for feeding. The orange jasmine trees were replaced with new ones every 4 days. Samples were collected at 3 d AAP, 6 d AAP, 12 d AAP, and 24 d AAP, with *n* = 6 × 10 psyllids per AAP for CTV detection.

### 2.7. Midgut Dissection and Transmission Electron Microscopic Observation

The transmission electron microscopy (TEM) (Model: FEI/Talos L120C, Thermo Fisher Scientific, OR, USA) was used to further analyze the CTV-infected midgut at the ultrastructural level. Samples were fixed with 2.5% (*v*/*v*) glutaraldehyde in 0.1 M of potassium phosphate buffer at PH 7.4 for 4 h at room temperature (RT) and then temporarily stored at 4 °C. After fixation, samples were rinsed with 0.1 M phosphate buffer (PB, pH = 7.4) four times. Postfixtion was then done by transferring samples into 2% osmium tetroxide (*w*/*v*) for another 2 h at RT and then in 1% uranyl dioxyacetate solution at 4 °C overnight. The samples were further rinsed with ddH_2_O four times at intervals of 10 min, dehydrated in 30%, 50%, 70%, and 90% ethanol successively for 10 min each, in 100% ethanol and then 100% acetone for 15 min twice, respectively. Samples were infiltrated and embedded in acetone resin solution (acetone: resin = 1:1) for 2 d, followed by overnight incubation in acetone resin solution (acetone: resin = 1:3). Sample sections were embedded with pure resin and mounted on 200-mesh form var-coated copper grids, sectioned at 60 nm thickness, viewed and photographed using the TEM.

### 2.8. Data Analysis

CTV and CLas concentrations (copy numbers) in samples of *D*. *citri* were calculated according to the standard curve. CTV and/or CLas carried by *D*. *citri* at different developmental stages were compared and analyzed. The results were statistically analyzed and diagram plotted using Microsoft Excel 2019, IBM SPSS Statistics 26, and Origin 2018. Kruskal–Wallis test, independent *t*-test with Dunn’s multiple comparisons, or the Student’s *t*-test was used for significant difference analysis of pathogen copy numbers in different *D*. *citri* populations (*p* < 0.05). Data were subjected to statistical analysis by one-way analysis of variance (ANOVA) followed by Duncan’s new multiple range test. Different letters in the figure indicate significant differences at *p* < 0.05 level.

## 3. Results

### 3.1. Incidence of CTV and CLas in D. citri from Citrus Orchards

**Figure 1:**
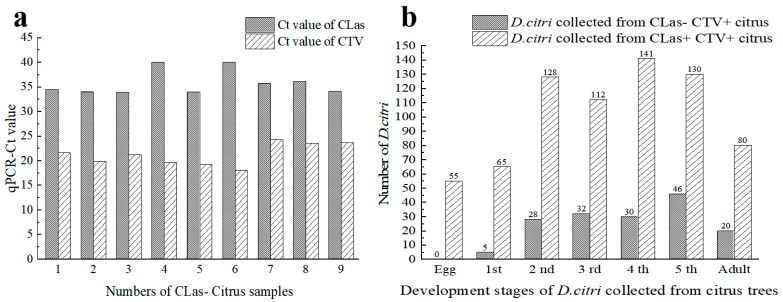
Detection results of ‘*Candidatus* Liberibacter asiaticus’ (CLas) and *Citrus tristeza virus* (CTV) in the *D*. *citri* samples from the trees in citrus orchards. (**a**) Detection results of CLas and CTV in branches of the nine citrus trees without HLB; (**b**) The number of *D*. *citri* carrying CLas or CTV at each developmental stage. CTV+: CTV positive; CLas+: CLas positive; CLas−: CLas negative. 1st, 2nd, 3rd, 4th, 5th indicate 1st instar, 2nd instar, 3rd instar, 4th instar, and 5th instar, respectively.

All field citrus plants used in this study were infected with CTV. Of the 40 trees in 3 orchards, 31 were detected as CLas-positive, and 9 were CLas-negative. The average Ct values of the 31 plants were 22.54 ± 0.48 for CLas detection and 22.20 ± 0.43 for CTV detection, respectively. While the average Ct values of CLas and CTV of the nine plants were 35.83 ± 0.93 and 21.22 ± 0.74, respectively (Figure 1a).

In total, 711 psyllid samples, including 55 eggs, 576 nymphs, and 80 adults (Table A2), were collected from the 31 CTV^+^CLas^+^ trees (Figure 1b). Neither CTV nor CLas were detected in the eggs, with 5 eggs pooled into 11 samples. The 1st instar nymphs did not carry CLas, but 38.46% of them were CTV-positive. For the 2nd to the 5th instar nymphs and adults, 61.25% to 85.71% of individuals were CTV-positive, while the CLas infection rates were relatively lower (from 28.13% for the 2nd instars to 60% for the adults). Relative high CTV-positive rates were observed in the 3rd and 4th instars. By contrast, the CLas–infection rates showed an increment with the age of the psyllid.

**Figure 2:**
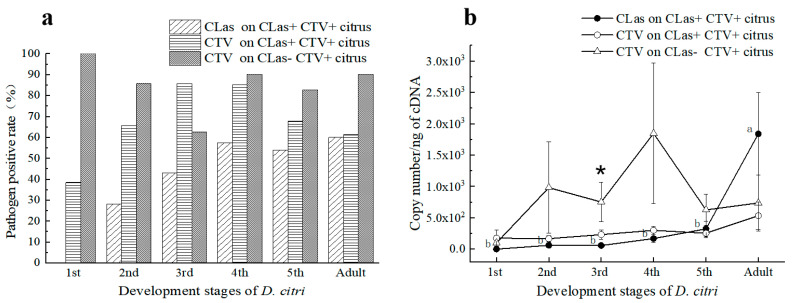
Presence and titers of ‘*Candidatus* Liberibacter asiaticus’ (CLas) and *Citrus tristeza virus* (CTV) in *D*. *citri* from CLas–infected and uninfected trees in citrus orchards. (**a**) Percentage of different stages’ *D*. *citri* that tested positive for CLas or CTV; (**b**) The average copy numbers of CLas or CTV carried by *D*. *citri* at different developmental stages; CLas+: CLas positive; CLas−: CLas negative; CTV+: CTV positive; CLas+CTV+ Citrus: Citrus plants infected by both CLas and CTV; CLas-CTV+ Citrus: Citrus plant infected by CTV but not by CLas. The data were subjected to statistical analysis by one–way analysis of variance (ANOVA) followed by Duncan’s new multiple range test. Different letters in the figure indicate significant differences at *p* < 0.05 level. *: Statistical significance in 95% confidence interval (*p* = 0.05) by independent sample *t*-test.

For the pathogen titers in the 711 psyllid samples, the average Ct values for CLas detection of CLas^+^ samples of 2nd to 5th instar nymphs and adults were 29.36 ± 0.62, 29.46 ± 0.52, 28.56 ± 0.42, 27.84 ± 0.37, and 26.51 ± 0.43, respectively. The Ct values of CLas^+^ adults were significantly lower (with CLas titers significantly higher) than those of the nymphs (*p* < 0.05) (Figure 2a). By contrast, no significant difference was in the CLas titers acquired by nymphs of different stages. Comparatively, the average Ct values of CTV^+^ samples of 1st–5th instar nymphs and adults were 29.90 ± 1.20, 29.46 ± 0.45, 29.20 ± 0.47, 28.40 ± 0.31, 29.02 ± 0.32, and 28.46 ± 0.36, respectively. The Ct values of the CTV^+^ 4th instar nymphs and adults were significantly lower than those of the 1st to 3rd instar nymphs and the 5th instar nymphs (*p* < 0.05). Likewise, there was no significant difference in the CTV concentration obtained by nymphs of different instars.

In total, 161 *D. citri* samples were collected from CLas^−^ CTV^+^ trees in the field. The CTV positive rates of 1st to 5th instars nymphs and adults are shown in Figure 2a. The mean Ct values of the CTV^+^ samples were 28.98 ± 0.00, 28.13 ± 1.45, 27.15 ± 1.39, 27.46 ± 1.11, 27.97 ± 0.56, and 28.60 ± 0.66, respectively. There was no significant difference in the average Ct value of CTV carried by the *D. citri* at different developmental stages. In general, the content of CTV contained by 1st to 4th instar *D. citri* increased with its developmental stages.

Comparatively, the titers of CTV acquired by the nymphs from the 2nd to 5th instars and adults from the CLas^−^CTV^+^ citrus trees were higher than those of the same developmental stages fed on the HLB-affected (CLas^+^CTV^+^) trees. Herein, the titers of CTV acquired by the 3rd instar nymphs feeding on CLas-negative trees were significantly higher than those of the 3rd instar nymphs feeding on CLas-infected trees (*p* < 0.05) (Figure 2b). Hence, we concluded that CLas in plants might inhibit CTV acquisition by *D. citri*.

### 3.2. CLas and CTV Acquisition by D. citri under Laboratory Conditions

#### 3.2.1. CLas and CTV Acquisition on Citrus Trees Infected by Both Pathogens

**Figure 3:**
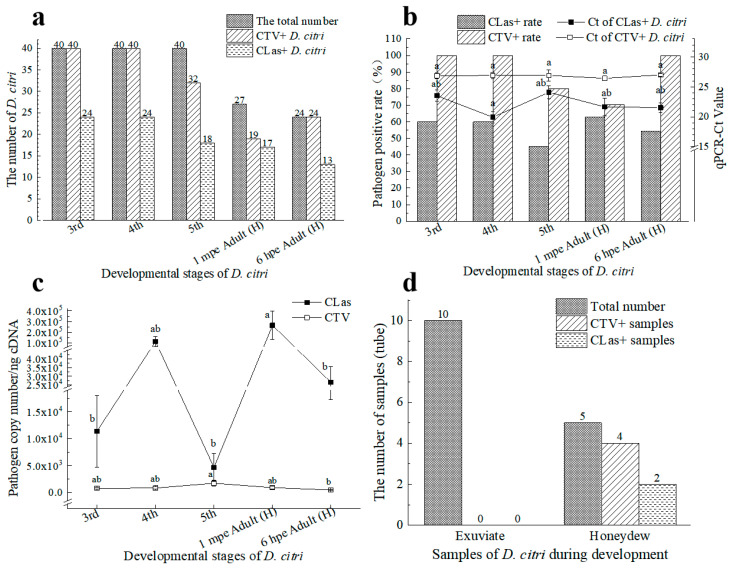
Acquisition of ‘*Candidatus* Liberibacter asiaticus’ (CLas) and *Citrus tristeza virus* (CTV) by *D*. *citri* from *Citrus reticulata* Blanco “Shatangju” carrying both CTV and CLas (CLas+CTV+) under experimental conditions. (**a**) The number of *D*. *citri* at different developmental stages carrying CLas or CTV; (**b**) Percentage of *D*. *citri* that tested positive for CLas or CTV at different developmental stages and the average Ct values of CLas or CTV of them; (**c**) The average copy numbers of CLas or CTV carried by *D*. *citri* at different developmental stages; (**d**) CLas and CTV-carrying status of exuviate and honeydew of *D*. *citri*. CLas+: CLas positive; CLas−: CLas negative; CTV+: CTV positive; 1 mpe Adult (H): *D*. *citri* adults that were 1 min after emergence on healthy *Citrus reticulata* Blanco “Shatangju”; 6 hpe Adult (H): *D*. *citri* adults that were 6 h after emergence on healthy *Citrus reticulata Blanco* ‘Shatangju’. Different letters in the figure indicate significant differences at *p* < 0.05 level.

After rearing from eggs to a population with all five stages’ nymph and adults on the CLas and CTV positive trees, pathogen acquisition results of 120 nymphs from 3rd instar to 5th instar (with 40 in each group) and 61 adults are shown in Figure 3a,b. The CLas positive rate of the 3rd to 5th instar nymphs was from 45.00% to 60%, respectively, with average Ct values of CLas^+^ samples of 23.52 ± 0.96, 19.93 ± 0.93, and 24.09 ± 1.06, respectively. The CTV positive rate of the 3rd to 5th instar nymphs were all higher than 80%, while the average Ct values of CTV^+^ samples were 26.85 ± 0.47, 26.91 ± 0.53, and 26.91 ± 0.94, respectively. There were 62.96% (Ct = 21.65 ± 1.47) and 70.73% (Ct = 26.43 ± 0.41) newly emerged adult individuals (recorded as 1 min post-emergence) found to be positive in CLas detection and CTV detection, respectively. After 6 h on CLas^−^CTV^−^ trees, 54.17% (Ct = 21.53 ± 0.78) and 100.00% (Ct = 26.99 ± 0.24) of them were positive for CLas and CTV, respectively. The *D. citri* kept carrying CTV and CLas after emergence on healthy ‘Shatangju’ plants. There was no significant difference between the average Ct values of CLas and CTV carried by all stages’ psyllids. No CTV particles were detectable in the exuviates, whereas they could be excreted, as the honeydew samples were detected as CTV-positive (Figure 3d).

#### 3.2.2. CLas Facilitated the CTV Acquisition by *D. citri* in the Early Stage and Inhibited the CTV Acquisition Later

**Figure 4:**
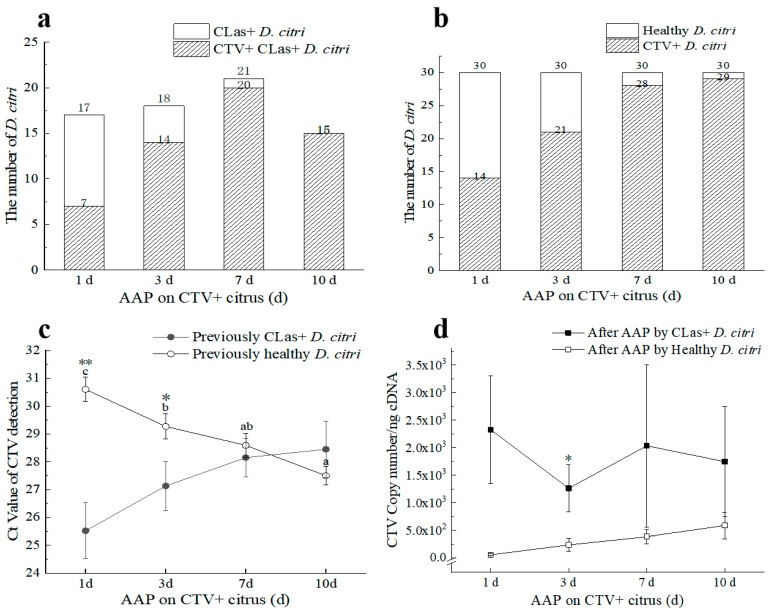
Effects of ‘*Candidatus* Liberibacter asiaticus’ (CLas) and *Citrus tristeza virus* (CTV) acquisition by different *D*. *citri* collected from CTV+ *Citrus grandis* Osbeck at 1–10 days after acquisition access period (AAP) in laboratory conditions. (**a**) The number of CTV-carrying *D*. *citri* after AAP of CLas+ *D*. *citri* population; (**b**) The number of CTV-carrying *D*. *citri* after feeding by healthy *D*. *citri* at different days’ AAP; (**c**) Ct values (mean ± SE) of CTV+ *D*. *citri* after different time’s AAP on CTV+ citrus by previously CLas+ or CLas− *D*. *citri* adults; (**d**) CTV titers in CTV+ *D*. *citri* of different AAP time by previously CLas+ or CLas− *D*. *citri*. CLas+: CLas positive; CTV+: CTV positive. Different letters in the figure indicate significant differences at *p* < 0.05 level by one–way analysis of variance (ANOVA) followed by Duncan’s new multiple range test. * or **: Independent sample *t*-test or Duncan’s Multiple range test was used to determine statistical significance in 95% confidence interval (*p* = 0.05) or 99% confidence interval (*p* = 0.01).

Two *D. citri* populations, namely CLas^+^CTV^−^ and CLas^−^CTV^−^, with 150 individuals of the same age in each population, were reared on the CTV-infected *Citrus grandis* Osbeck for CTV acquisition. Adding together, 17, 18, 21, and 15 *D. citri* adults were detected as CLas-positive at 1 d, 3 d, 7 d, and 10 d AAP (Figure 4a). Of them, the proportion of CTV-positively increased over time, i.e., 41.18% (Ct = 25.52 ± 1.00), 77.78% (Ct = 27.13 ± 0.88), 95.24% (Ct = 28.15 ± 0.69), and 100.00% (Ct = 28.45 ± 1.02), respectively. Similarly, the rates of CTV acquisition increased with time for the other *D. citri* population (Figure 4b,c). The proportion of CTV-positive adults at 1 d, 3 d, 7 d, and 10 d AAP for previously CLas^−^CTV^−^ *D. citri* were 46.67% (Ct = 30.60 ± 0.44), 70.00% (Ct = 29.27 ± 0.46), 93.33% (Ct = 28.59 ± 0.43), and 96.67% (Ct = 27.50 ± 0.33), respectively.

Collectively, the CTV titers increased over time in the 10 d acquisition period for CLas^−^ *D. citri* but not for the CLas^+^ *D. citri*. CTV concentration acquired by CLas^−^ *D*. *citri* was significantly higher at 10 d AAP compared with those of 1 d and 3 d AAPs according to the Ct values (Figure 4c). However, the abundance of CTV acquired by the CLas^+^ *D. citri* was higher than that by the CLas^−^ *D. citri*, especially when adults were given access to the CTV^+^ plants for 3 days (*p* < 0.05) (Figure 4d). This result suggests that CLas facilitated the CTV acquisition by *D. citri* in the early stage (Figure 4d). However, the CTV acquisition efficiency was inhibited thereafter.

#### 3.2.3. CLas Acquisition by *D. citri* was Promoted by CTV

After 15-day AAP of CTV, the 3rd instar *D. citri* nymphs had emerged to adults for about one week. This psyllid population (CTV^+^CLas^−^) was tested 100% (*n* = 20) positive for the presence of CTV (Ct = 25.54 ± 0.28). The population and the CTV^−^CLas^−^ population of the same age were placed on CLas^+^CTV^−^ Tanaka seedlings for CLas acquisition. Noteworthily, although feeding on the CTV-free trees, the CTV titers in the first population remained at a stable level during the 10-day CLas acquisition.

**Figure 5:**
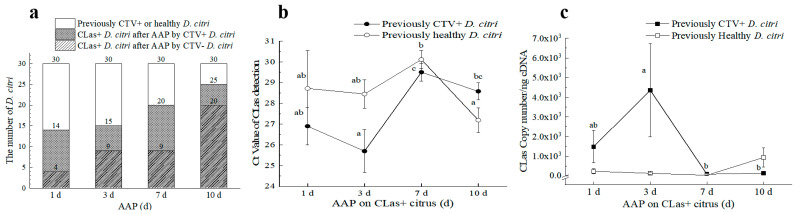
Effects of *Citrus tristeza virus* (CTV) on ‘*Candidatus* Liberibacter asiaticus’ (CLas) acquisition on CLas–carrying *Citrus tangerine* Hort. Ex Tanaka by previously CTV+CLas− or CTV−CLas− *D*. *citri* adults. (**a**) The number of CLas+ *D*. *citri* after different AAPs; (**b**) The average Ct value of CLas carried by the two *D*. *citri* populations after feeding; (**c**) Comparison of CLas content between CTV+ *D*. *citri* and healthy *D*. *citri* after different feeding days. CLas+: CLas positive; CTV+: CTV positive; CTV−: CTV negative; Different letters in the figure (**b**,**c**) indicate significant differences at *p* < 0.05 level.

CLas-positive rates increased for both *D. citri* populations in the 10 d AAP period. Wherein the rates of CLas^+^ psyllids in the CTV^+^CLas^−^ *D. citri* population at all four testing stages (From 46.67% to 83.33%) were all higher than those in the CTV^−^CLas^−^ *D. citri* population (from 13.33% to 66.67%) (Figure 5a). Additionally, a significantly higher level of CLas was acquired at the early stage (3 d AAP) than that of the later stage (7 d AAP and 10 d AAP) by the CTV^+^ psyllid (Figure 5b,c). Although relatively lower Ct values for CLas detection were detected for the CTV^−^ psyllid at 10 d following CLas AAP, there was no significant difference among the testing stages in CLas titers within them. When comparing the effect of CLas acquisition between the two psyllid groups, CTV^+^ *D. citri* adults acquired much more CLas at 1 d AAP and 3 d AAP. However, the differences were not significant after 7 d AAP. Consequently, the CTV^+^ psyllid was more efficient in CLas acquisition according to CLas-positive proportion and CLas titers.

#### 3.2.4. CTV Contributed to the Transmission of CLas by *D. citri*

Four groups of the same aged *D. citri*, namely CLas^+^CTV^−^, CLas^+^CTV^+^, CLas^−^CTV^+^, and CLas^−^CTV^−^, were used for the CTV and CLas transmission. For the first and second groups, the CLas-positive proportions were 50%, while for the second and third groups, the CTV-positive rates were 90%. All selected “Shatangju” trees used for transmission were confirmed to be negative in CTV and CLas after detecting by RT–qPCR.

**Figure 6:**
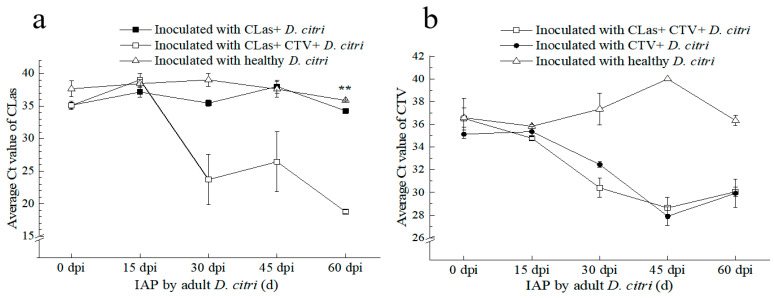
Transmission effect of ‘*Candidatus* Liberibacter asiaticus’ (CLas) and/or *Citrus tristeza virus* (CTV) by *D*. *citri* to *Citrus reticulata* Blanco “Shatangju” plants. (**a**) Ct values of CLas detection in “Shatangju” samples after inoculation access period (IAP) by *D*. *citri* carrying different pathogens. (**b**) Ct values of CTV detection in “Shatangju” plants after IAP by the *D*. *citri* carrying different pathogens. CLas+: CLas positive; CTV+: CTV positive; CLas+ & CTV+: CLas positive and CTV positive. **: Independent sample *t*-test and Duncan’s Multiple Range test to determine statistical significance within a 99% confidence interval (*p* = 0.01).

Firstly, we compared the influence of CTV in psyllid on CLas transmission. All plants tested positive for CLas at 30 and 60 days after IAP by the CLas^+^CTV^+^ adult *D. citri*. The average Ct value for the CLas detection was 23.70 ± 3.88 at 30 d after IAP. Moreover, the mean Ct values of plants at 45 d and 60 d IAPs were significantly lower than those feeding–inoculated by the CLas^+^CTV^−^ *D. citri* adults. These results suggest that *D. citri* carrying CTV can transmit CLas to “Shatangju” plants more quickly, and CLas^+^CTV^+^ *D. citri* transmitted more CLas inoculum (Figure 6a).

Secondly, the influence of CLas in psyllid on CTV transmission was analyzed. As shown in Figure 6b, the two groups of 1-week-post-emergence *D. citri* (CLas^+^CTV^+^ and CLas^−^CTV^+^) transmitted the virus to healthy ‘Shatangju’ plants at a rate of 100% after 30 days following the IAP. The average Ct values of CTV detection by RT–qPCR were 30.40 ± 0.84 and 32.45 ± 0.24, respectively, for the trees fed by the two psyllid populations at 30 d after IAP, showing no significant difference. These results indicated that the CLas carried by psyllid had no significant effect on the efficiency of CTV transmission to *Citrus reticulata* Blanco ‘Shatangju’.

**Figure A1:**
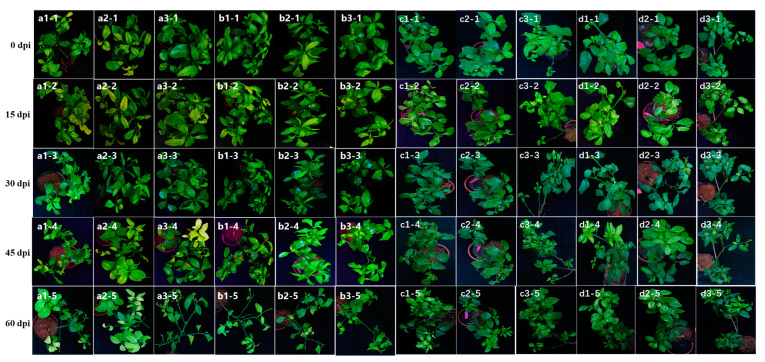
Symptoms of *Citrus reticulata* Blanco “Shatangju” at different periods after inoculation by CLas+ *D. citri*, healthy *D. citri*, CLas+ CTV+ *D. citri,* and CTV+ *D. citri*. a, “Shatangju” plants inoculated by CLas^+^ *D. citri*; b, Plants inoculated by healthy *D. citri*; c, Plants inoculated by CLas^+^ CTV^+^ *D. citri*; d, Plants inoculated by CTV^+^ *D. citri*; Three replicates were set for each experimental treatment; dpi: days post-inoculation.

On all ‘Shatangju’ plants fed by *D. citri*, the leaves curled and showed less luster after IAP (recorded as 0 dpi) (Figure A1 a1–1 to d3–1). The leaves were slightly withered at 0–30 dpi, with some falling off. However, the situation was improved after the booting of new shoots (Figure A1 a1–1 to d3–3). After inoculation by the CLas^+^ *D. citri*, the leaves of the new shoots of “Shatangju” showed slight chlorosis at 15 dpi, although no CLas was detected until 60 dpi. No blotchy mottled leaves were observed even at 60 dpi (Figure A1 a1–2 to a3–5). With both pathogens at a detectable level from 30 to 60 dpi by CLas^+^ CTV^+^ *D. citri*, the young leaves of new shoots showed no typical symptoms of HLB or apparent variegated yellowing. However, the number of new shoots decreased (Figure A1 c1–3 to c3–5). Although no obvious symptoms of recession or dwarfing were observed, the mature leaves of CTV–positive “Shatangju” trees were slightly yellow in veins, cured, and fell easily (Figure A1 c1–3 to d3–5).

### 3.3. Ultrastructure of CTV in Midgut

**Figure 7:**
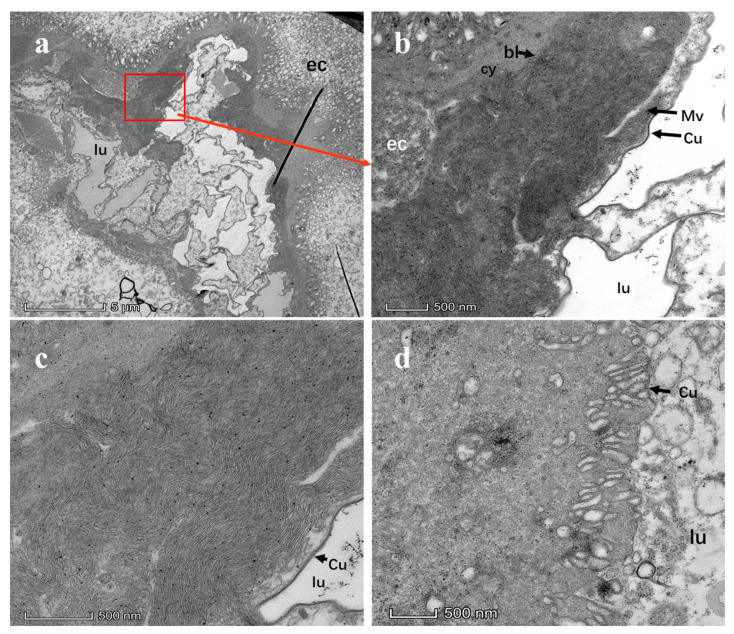
Transmission electron microscopy images of *D*. *citri* midguts with and without *Citrus tristeza virus* (CTV). (**a**) TEM images of *D*. *citri* midguts carrying CTV at a magnification of 2600; (**b**) TEM images of midguts of *D*. *citri* carrying CTV at a magnification of 13500; (**c**) TEM images of midguts of *D*. *citri* carrying CTV at a magnification of 22000; (**d**) TEM images of midguts of healthy *D*. *citri* at a magnification of 22,000. Lu: lumen; Cy: cytoplasm; ec: epithelial cell; bl: basal lamina; mv: microvilli; Va: Cytoplasmic vacuoles; Cu: cuticle.

**Figure A2:**
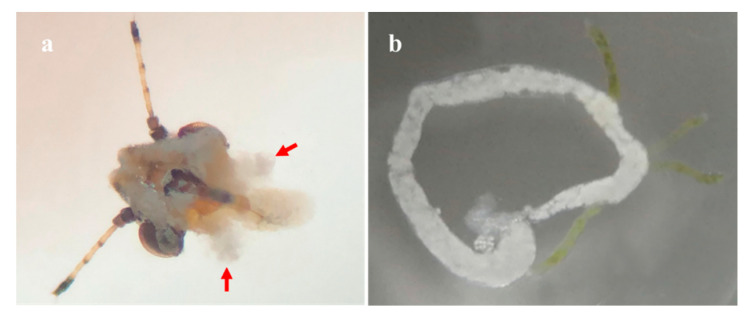
Anatomical photographs of salivary glands and midguts of adult *D. citri*. (**a**) salivary glands of *D. citri*; (**b**) Midguts of *D. citri*. The red arrow points to the tissue of the salivary gland.

After assessing the CTV distribution in salivary glands and midguts using CTV-positive *D*. *citri* adults, we confirmed that CTV titers in the midguts of adult *D. citri* were significantly higher than those in salivary glands (*p* < 0.05). This indicates CTV can penetrate the inner body of *D*. *citri* adults. Therefore, the midguts of adult *D. citri* were selected as the experimental samples for subsequent TEM observation of CTV (Figure A2). In total, 30 CTV-positive adult *D. citri* and 15 healthy adult *D. citri* were collected, and their midguts were dissected for TEM assay. A large number of long linear CTV particles were observed between microvilli and basal layer in TEM slices of midgut tissues of CTV-carrying adults (Figure 7a–c). Comparatively, no apparent CTV morphology was observed in slices of midgut tissues of healthy adults (Figure 7d).

### 3.4. CTV Replicates in Midgut of D. citri

CTV could be detected at the midgut of 10% of adult *D*. *citri* individuals as early as 3 h AAP. The proportion of midgut samples detected as CTV-positive increased with confinement time, with 20%, 40%, 50%, and 80% at 6 h AAP, 24 h AAP, 48 h AAP, and 72 h AAP, respectively. The mean Ct values of the detected samples decreased gradually from 35.58 ± 1.22 at 3 h AAP to 28.43 ± 0.8 at 72 h AAP. The CTV titers at 72 h AAP were significantly higher than those before 48 h AAP (*p* < 0.001). After the *D*. *citri* population was transferred onto the CTV^−^ orange jasmine seedlings, CTV average Ct values at 3 d AAP, 6 d AAP, 12 d AAP, and 24 d AAP were 26.84 ± 1.93, 27.93 ± 1.95, 28.80 ± 1.36, 28.60 ± 1.37, and 28.57 ± 1.26, respectively. No significant difference was observed neither among the average Ct values nor among the average copy numbers of CTV at different AAPs in CTV persistence. This indicates if CTV was transmitted by *D*. *citri*, the transmission mode would be circulative propagative.

## 4. Discussion

In this study, it was speculated that CTV could penetrate (Section 3.3) and replicate (Section 3.4) in the body of *D. citri*, which was different from the non-circulating semipersistent manner of aphids [16]. Further research is required to determine when or whether CTV moves from the gut to the salivary glands to become circulative. As CTV particles were enriched in midguts of *D*. *citri*, and there was no significant decrease of CTV titers during the 24-day persistence assay, the CTV transmission manner by *D*. *citri* was suggested to be the same as that for CLas transmission [37,38]. Although the conclusion that CTV would be transmitted by *D. citri* is still controversial, this does not hinder us from exploring the details of CTV-vector-CLas interaction.

Co-infection of CTV and CLas in the field trees was found to be common in this study and several previous studies [39,40,41,42]. Both CTV and CLas were vector-transmitted obligate pathogens of phloem, although they belong to bacteria and viruses, respectively. Most natural vector insects, such as *Trialeurodes vaporariorum*, *Delphacodes kuscheli,* and *Bemisia tabaci,* can carry, acquire and even transmit more than one virus, which causes co-infection of host plants [43,44,45,46,47]. *D*. *citri* is a pest feeding on phloem sap by piercing mouthparts. Hence, finding the co-infection status of the two pathogens in *D*. *citri* from the field is not surprising. Although the results presented herein showed higher CTV titers could be detected in the plant host than those in the vectors, CTV was found accumulated from nymphs to adults when feeding on CTV^+^ citrus plants. The results strongly suggest differential adaptation of these pathogens to the *D. citri* or differential adaptation of CTV to different hosts. In this study, the CTV-carrying rates of *D. citri* were higher than CLas-carrying rates in all developmental stages on the citrus plant co-infected by two pathogens. Combined with the previous studies [8,33,48,49], this difference indicated that *D. citri* was more likely to acquire and carry CTV from host plants compared to acquiring CLas, although with lower titers.

Vector-born disease epidemiology is impacted by interactions between pathogens and the pathogen accumulation efficiency in host plants [50]. In the field trial, we also found that the CLas in plants would inhibit CTV acquisition by *D. citri*. After being infected by insect-borne pathogens, pigments, nutrients, hormones, volatile organic compounds, and resistance levels of host plants change to varying degrees, thereby affecting the behavior and adaptability of vector insects and thus affecting the spread and prevalence of other pathogens [51,52,53]. On the one hand, the content of volatile organic compounds produced by citrus due to the infection of CLas may reduce the attraction of citrus plants to *D. citri* [54,55]. On the other hand, when citrus was infected with CLas, the phloem autoimmune response enhanced [56], thus influencing the CTV acquisition by *D. citri*. Whether changes in phloem cells or volatiles of citrus co-infected by CLas and CTV influence the attractiveness of host plants to the vector remains to be further explored scientifically.

In the vectors capable of acquiring or transmitting more than one pathogen, the acquisition or transmission of one pathogen may be influenced by the other pathogen. We speculated that CTV infection promoted the feeding of *D. citri* on HLB trees and alerted the acquisition and transmission of CLas (Section 3.2.2 and Section 3.2.3). The relationship between the vector and the pathogen can significantly impact transmission efficiency. For example, the *Tomato infectious chlorosis virus* is much more efficiently transmitted by *Trialeurodes vaporariorum* than the *Tomato chlorosis virus* [56]. Similarly, Glaser et al. [57] proposed that bacterial infection of vector insects can enhance their resistance to viruses. Zouache et al. [58] found that secondary symbiotic bacteria levels in the body of insect vectors can be affected after being infected by a virus. Yan [59] proposed that pathogens could directly or indirectly change the feeding behavior of vector insects to promote their effective transmission. This study preliminarily verified the interaction between CLas and CTV in *D. citri*. Studying the relationship between plant viruses and bacteria in vector insects can provide an important reference for revealing the internal mechanism of pathogen transmission by vector insects that can carry two or more pathogens.

It should be noted that for the field and laboratory CLas/CTV carrying/acquisition assays, different stages’ *D. citri* samples were collected simultaneously. To be rigorous, *D. citri* populations carrying different pathogens in different stages should be used for the pathogen acquisition, persistence, and transmission study to better elucidate this interaction among *D. citri*-CTV-CLas. Besides, the effects of CTV infection on vector–plant interaction, including development, host selection, and feeding behavior, are being studied in our lab. It is clear that the CTV-infected *D. citri* adults preferred to feed on citrus leaves infected by CLas. A direct–current electrical penetration graph (DC–EPG) study has suggested that penetration in the CTV^+^ phloem was much easier than in the CTV^−^ phloem. Further studies should determine whether CTV infection alters the attractiveness of the host plant to the vector and how the CTV affects the behavioral response of its vector from the molecular level.

## 5. Conclusions

In this study, the characteristics of CLas and CTV acquisition and transmission by *D. citri* were systematically analyzed for the first time based on the previous confirmation that *D. citri* would acquire and persist CTV. The results presented herein further suggested that the proliferation of CLas and CTV in *D. citri* was similar. The titers of CLas carried by adult *D. citri* were higher than those of nymphs, but the titers of CTV carried by the adult *D. citri* were not significantly different from those of the nymphs. CTV carried by *D. citri* is beneficial to the acquisition and transmission of CLas of this vector. This study combines CTV with the research hotspots of the ‘citrus–*D. citri*–CLas’ interactions raise essential scientific questions for further research on the molecular mechanism of pathogen transmission by *D. citri* and provide new ideas for the comprehensive prevention and control of HLB and Citrus tristeza disease.

## Figures and Tables

**Table 1:** Information of citrus seedlings grafted for CLas or CTV acquisition by *D. citri*.

Citrus Cultivars	Pathogen Carried by Scions	Ct Value of CLas	Ct Value of CTV
*Citrus reticulata* Blanco “Shatangju”	CLas and CTV	24.27 ± 1.01	22.17 ± 0.44
*Citrus tangerine* Hort. Ex Tanaka	CLas	23.21 ± 0.83	35.49 ± 0.72
*Citrus grandis* Osbeck	CTV	37.41 ± 1.44	23.01 ± 1.22

The CTV was *Citrus tristeza virus* strain CT31, kindly provided by Yan Zhou from Citrus Research Institute of China, and the CLas was ‘*Candidatus* Liberibacter asiaticus’ strain A4 from Citrus Huanglongbing Research Laboratory in South China Agricultural University. Both CT31 and A4 were on sour orange (*Citrus aurantium* L.) plants.

## Data Availability

The data presented in this study are available on request from the corresponding authors.

## Appendix A

**Table A1:** Information on plants collected from three citrus orchards in Guangdong Province.

Orchard Number	Sampling Time	Location	Orientation Information	Age of the Tree/Year-Old	Citrus Cultivars	Number of Trees Sampled
1	August, 2020	Zhanjiang	21°26′20.23″ N, 110°28′02.09″ E	2	*Citrus sinensis* Osbeck. ‘Hongjiangcheng’	10
2	November, 2020	Deqing	23°13′21.56″ N, 111°53′57.89″ E	10	*Citrus reticulata* Blanco var. gonggan	14
3	November 2020	Deqing	23°19′21.91″ N, 112°12′16.52″ E	4	*Citrus medica* L. var. sarcodactylis Swingle	16

Orchard numbers are the same as those in Table 1.

**Table A2:** Information on *D. citri* samples collected from three citrus orchards.

Orchard Number	Developmental Stages of *D. citri* (Quantity/Individual)						
	Egg	1st	2nd	3rd	4th	5th	Adult
1	45	30	20	8	24	78	37
2	0	30	108	104	135	88	37
3	10	10	28	32	12	10	26
Total	55	70	156	144	171	176	100

Orchard numbers are the same as those in Table 1. Egg, 1st, 2nd, 3rd, 4th, 5th, and Adult indicate the developmental stages of Egg, 1st instar nymph, 2nd instar nymph, 3rd instar nymph, 4th instar nymph, 5th instar nymph, and adult, respectively.
